# Native T1 Mapping and Clinical Risk Characterization in Non-Ischemic Dilated Cardiomyopathy: A Cardiac Magnetic Resonance Study

**DOI:** 10.3390/jcdd13060279

**Published:** 2026-06-19

**Authors:** Manuela Montatore, Marco Rella, Eleonora Indolfi, Federica Masino, Ruggiero Tupputi, Eluisa Muscogiuri, Giuseppe Guglielmi

**Affiliations:** 1 Department of Clinical and Experimental Medicine, School of Medicine, University of Foggia, 71122 Foggia, Italy; manuela.montatore@unifg.it (M.M.); federicamasino@gmail.com (F.M.); 2 Radiology Unit, “A. Perrino” Hospital, ASL Brindisi, 72100 Brindisi, Italy; marcorella91@yahoo.it (M.R.); eluisa.muscogiuri@asl.brindisi.it (E.M.); 3 Cardiology Unit, “D. Camberlingo” Hospital, ASL Brindisi, 72021 Francavilla Fontana, Italy; eleonoraindolfi@gmail.com; 4 Radiology Unit, “Dimiccoli” Hospital, Viale Ippocrate 15, 70051 Barletta, Italy; rutudott@gmail.com

**Keywords:** cardiac magnetic resonance, native T1 mapping, dilated cardiomyopathy, late gadolinium enhancement, myocardial fibrosis, risk stratification, prognosis

## Abstract

**Background:** Risk stratification in non-ischemic dilated cardiomyopathy (DCM) remains challenging because left ventricular ejection fraction (LVEF) and late gadolinium enhancement (LGE) do not fully capture the underlying myocardial substrate. Septal native T1 mapping provides a quantitative assessment of diffuse myocardial abnormalities and may contribute to myocardial tissue characterization within a multiparametric CMR framework. **Methods:** This retrospective single-center study included 45 consecutive patients with non-ischemic DCM referred for clinically indicated CMR at Perrino Hospital, Brindisi, Italy, between November 2023 and November 2025. All examinations were performed using a standardized CMR protocol including cine imaging, LGE, and native T1 mapping on a 1.5-T Siemens Healthineers scanner. Septal native T1 was used as the primary mapping parameter because of its established reproducibility and robustness for myocardial tissue characterization. Patients were followed for a composite endpoint including all-cause mortality, major ventricular arrhythmic events, appropriate ICD therapy, and hospitalization for heart failure. Endpoint coding was verified, and all analyses were performed using the final validated dataset. **Results:** During a median follow-up of 15 months, 14 patients (31.1%) experienced the composite endpoint. Patients with events had lower LVEF (27.1 ± 7.8% vs. 48.3 ± 10.5%; *p* < 0.001), higher LVEDVi (142.6 ± 28.5 vs. 110.6 ± 23.4 mL/m^2^; *p* = 0.001), and higher septal native T1 values among patients with available T1 measurements (1047.5 ± 25.0 vs. 1031.5 ± 24.3 ms; *p* = 0.065). ROC analysis identified a septal native T1 threshold of 1042 ms for prediction of the composite endpoint, with an exploratory AUC of 0.70. Event-free survival was lower in patients with septal native T1 ≥ 1042 ms. Given the limited number of events, all regression and hierarchical analyses should be interpreted as exploratory and hypothesis-generating. **Conclusions:** Higher septal native T1 values were observed in patients experiencing adverse clinical outcomes; however, native T1 was not independently associated with the composite endpoint in exploratory Cox regression analyses.

## 1. Introduction

Dilated cardiomyopathy (DCM), particularly in its non-ischemic form, represents a major cause of heart failure, ventricular arrhythmias, and sudden cardiac death. Despite advances in medical and device therapy, prognostic assessment in non-ischemic DCM remains incomplete. This is partly because the disease is biologically heterogeneous: different etiological substrates may converge toward a shared phenotype characterized by ventricular dilatation, systolic dysfunction, focal replacement fibrosis, diffuse interstitial remodeling, and arrhythmic vulnerability [1,2,3,4].

Current clinical risk stratification still relies predominantly on left ventricular ejection fraction (LVEF), functional status, and selected clinical variables. LVEF remains central to therapeutic decision-making, including consideration of implantable cardioverter-defibrillator (ICD) implantation for primary prevention. However, LVEF is an imperfect surrogate of risk. Adverse events may occur in patients with moderately reduced or even near-preserved systolic function, whereas many patients with severely reduced LVEF remain clinically stable. Therefore, risk in non-ischemic DCM should not be conceptualized exclusively as a function-based construct [5].

Cardiac magnetic resonance (CMR) is the reference imaging modality for the comprehensive evaluation of cardiomyopathies. It provides a highly reproducible assessment of ventricular volumes, systolic function, myocardial mass, and tissue characteristics. Late gadolinium enhancement (LGE) imaging allows detection of focal myocardial fibrosis and replacement scar, and its presence, extent, location, and pattern have been associated with adverse outcomes in non-ischemic DCM. Septal mid-wall LGE and more complex scar distributions have been linked to increased arrhythmic and mortality risk. However, LGE is intrinsically limited by its reliance on relative signal differences and is less sensitive for diffuse interstitial abnormalities, particularly when no normal reference myocardium is available [6,7,8].

**Figure 1:**
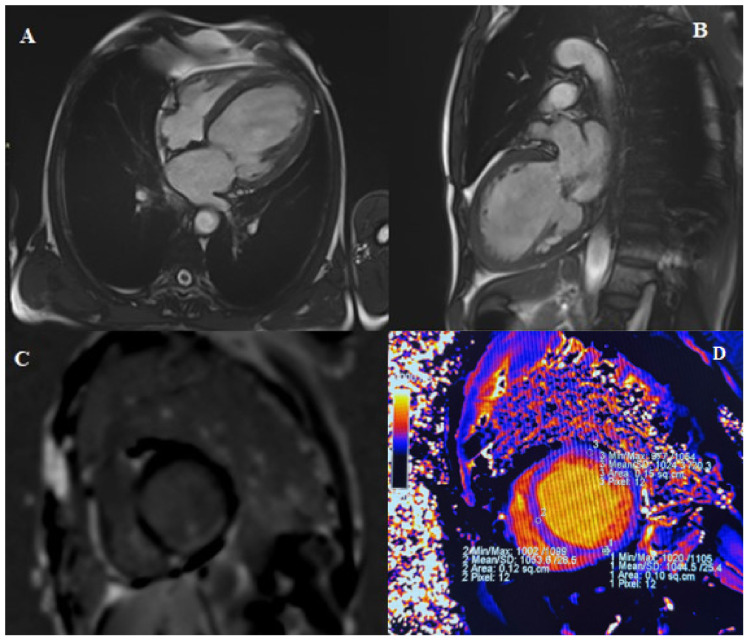
Representative multiparametric CMR findings illustrating focal scar-like enhancement in a patient evaluated for dilated cardiomyopathy. (**A**) Cine bSSFP four-chamber view showing left ventricular dilatation with reduced systolic function. (**B**) Cine bSSFP two-chamber view confirming global LV enlargement and impaired contractility. (**C**) Short-axis PSIR images demonstrating subendocardial enhancement involving the basal-to-mid inferior wall, with approximately 50% transmural extent. Given the ischemic-like appearance, significant coronary artery disease was excluded before classification within the non-ischemic DCM cohort. (**D**) Native T1 mapping (pre-contrast) with region-of-interest (ROI) analysis showing mildly elevated global myocardial T1 values (approximately 1042 ms), with lower septal values (~1010 ms), suggesting relatively limited diffuse myocardial involvement compared to focal fibrosis. This pattern highlights the predominance of focal replacement fibrosis. Dashed line indicates the local upper reference limit for native T1 (1017 ms; institutional reference value: 970 ± 47 ms).

**Figure 2:**
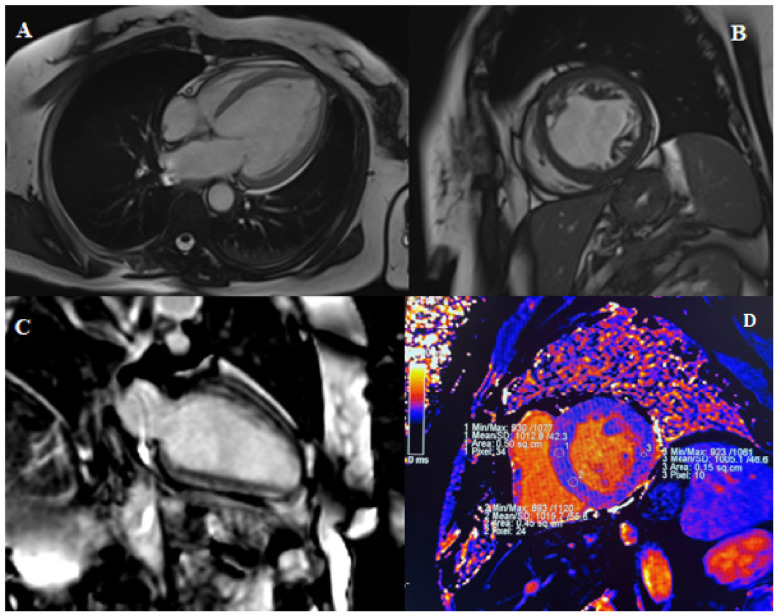
Representative CMR findings in a patient with non-ischemic dilated cardiomyopathy and predominant diffuse myocardial involvement. (**A**) Cine bSSFP four-chamber view showing left ventricular dilatation with mildly to moderately reduced systolic function. (**B**) Cine bSSFP short-axis view illustrating global ventricular remodeling. (**C**) Phase-sensitive inversion recovery (PSIR) two-chamber view demonstrating intramyocardial late gadolinium enhancement located in the anterior interventricular septum, consistent with non-ischemic fibrosis. (**D**) Native T1 mapping (pre-contrast) with ROI analysis showing borderline-high myocardial T1 values (global native T1 approximately 1005 ms; septal native T1 approximately 1010 ms) relative to the local reference range (970 ± 47 ms). This pattern illustrates the potential complementary value of quantitative mapping in characterizing diffuse myocardial abnormalities beyond focal LGE.

Native T1 mapping offers a complementary approach to myocardial tissue characterization. By quantifying intrinsic myocardial relaxation properties before contrast administration, native T1 mapping may detect diffuse myocardial abnormalities related to interstitial expansion, fibrosis, edema, and myocyte injury. In non-ischemic DCM, where diffuse myocardial remodeling may precede or coexist with focal replacement fibrosis, native T1 may provide biologically relevant information alongside LVEF and LGE (Figure 1 and Figure 2).

This study aimed to evaluate the association between septal native T1 values and adverse clinical outcomes in patients with non-ischemic DCM undergoing clinically indicated CMR, and to explore the potential role of septal native T1 within a multiparametric CMR risk characterization framework including LVEF and LGE [9,10].

## 2. Materials and Methods

### 2.1. Study Population

This retrospective, single-center observational study included consecutive patients with non-ischemic DCM referred for clinically indicated CMR at Perrino Hospital, Brindisi, Italy, between November 2023 and November 2025. During the study period, the center performed approximately 850–1000 clinically indicated cardiac CMR examinations, based on institutional activity records.

Non-ischemic DCM was defined as left ventricular systolic dysfunction and/or left ventricular dilatation in the absence of significant coronary artery disease or an ischemic CMR pattern. Significant coronary artery disease was defined as ≥50% luminal stenosis on invasive coronary angiography or coronary computed tomography angiography, when available. In patients without invasive or CT coronary assessment, an ischemic etiology was considered unlikely in the absence of infarct-pattern LGE and according to the clinical assessment of the referring cardiologist. LGE pattern was categorized using a predefined classification system as follows:

0 = absent;

1 = mid-wall;

2 = subepicardial;

3 = insertion-point;

4 = mixed/non-ischemic;

5 = ischemic pattern.

LGE location was defined according to the predominant segmental distribution:

0 = absent;

1 = septal;

2 = lateral;

3 = inferior;

4 = anterior;

5 = multiple locations.

LGE was assessed qualitatively according to its presence, predominant pattern, and anatomical distribution. Because quantitative LGE extent was not consistently available across the entire cohort, it was not incorporated into the primary prognostic analyses. Exclusion criteria were ischemic cardiomyopathy or infarct-pattern LGE, acute or subacute myocarditis, hypertrophic cardiomyopathy, arrhythmogenic cardiomyopathy, infiltrative cardiomyopathy, moderate-to-severe primary valvular disease, inadequate image quality, incomplete CMR protocol, unavailable native T1 mapping, and duplicate examinations. For patients with more than one CMR examination, only the first study was retained.

The final study population included 45 patients. Native T1 values were interpreted according to the local scanner- and sequence-specific reference range used at our center: 970 ± 47 ms. The local upper reference limit of 1017 ms was derived from the institutional normal reference value (970 ± 47 ms), corresponding to the mean plus one standard deviation.

The study was conducted in accordance with the principles of the Declaration of Helsinki. According to local regulations, formal ethical review and approval were not required for this retrospective study based on fully anonymized data. Patient consent was waived due to the retrospective nature of the study and anonymized data handling. All variables were collected using a structured database with anonymized alphanumeric identifiers (STUDY ID) to ensure data protection.

Categorical variables were encoded using binary or ordinal coding systems for statistical analysis, as follows:

- Sex: 0 = female, 1 = male
- Inpatient_outpatient: 0 = outpatient, 1 = inpatient
- Cardiovascular risk factors (hypertension, diabetes, dyslipidemia, smoking): 0 = absent, 1 = present
- Syncope and NSVT at baseline: 0 = absent, 1 = present
- LGE presence: 0 = absent, 1 = present
- Clinical endpoints: 0 = no event, 1 = event

NYHA functional class was recorded as an ordinal variable (I–IV). Continuous variables included:

- LVEF (%)
- LVEDVi and LVESVi (mL/m^2^)
- LV mass index (g/m^2^)
- RVEF (%)
- Native T1 values (ms), both global and septal when available.

### 2.2. CMR Acquisition

All CMR examinations were performed using a clinical 1.5-T scanner (MAGNETOM Aera, Siemens Healthineers, Erlangen, Germany), according to a standardized institutional protocol. The protocol included balanced steady-state free precession cine sequences for assessment of biventricular volumes and function, LGE imaging acquired 10–15 min after intravenous administration of a gadolinium-based contrast agent according to local clinical practice, and native T1 mapping performed before contrast administration using a modified Look-Locker inversion recovery sequence.

Cine images were acquired in standard long-axis views and in contiguous short-axis slices from base to apex. LGE images were acquired in matching long-axis and short-axis planes. Native T1 maps were acquired in short-axis orientation. Global and septal native T1 values were recorded when available.

### 2.3. CMR Analysis

CMR analyses were performed using ARGUS post-processing software (Siemens Healthineers, Erlangen, Germany) by experienced readers blinded to clinical outcomes. Left ventricular end-diastolic volume index (LVEDVi), left ventricular end-systolic volume index (LVESVi), left ventricular mass index, LVEF, and right ventricular ejection fraction (RVEF) were quantified from cine images using manual contouring and indexed to body surface area when appropriate [9,10,11,12].

LGE images were visually assessed for the presence or absence of focal myocardial fibrosis. LGE was considered present only when visible in appropriate imaging planes and not restricted to isolated right ventricular insertion-point enhancement. When present, LGE was characterized according to its predominant pattern and location, including mid-wall, subepicardial, focal, mixed, septal, free wall/lateral, inferior, or multiple distributions [13]. LGE was assessed qualitatively according to its presence, predominant pattern, and anatomical distribution. Because quantitative LGE extent was not consistently available across the entire cohort, it was not incorporated into the primary prognostic analyses. Consequently, LGE was analyzed as a binary variable (present/absent) for statistical purposes [14,15,16].

Septal native T1 was selected as the primary mapping parameter because septal measurements are less susceptible to partial-volume effects, motion artifacts, and regional heterogeneity, and have demonstrated higher reproducibility across studies. Native T1 values were obtained by placing regions of interest within the mid-interventricular septum while carefully avoiding the blood pool, focal artifacts, and areas of visible LGE when present. In accordance with current CMR recommendations, septal native T1 was considered the most robust and reproducible marker for the primary analyses.

### 2.4. Clinical Data and Follow-Up

Baseline clinical data were obtained from electronic medical records and included age, sex, inpatient or outpatient status, cardiovascular risk factors, NYHA functional class, syncope, non-sustained ventricular tachycardia (NSVT), and ICD-related variables when available.

Follow-up data were obtained from hospital records, outpatient evaluations, and, when necessary, direct contact with patients or referring physicians. Patients were followed from the date of the index CMR examination until the first clinical event or last available follow-up. Follow-up duration was calculated in months.

### 2.5. Study Endpoints

The primary endpoint was a composite of all-cause mortality, major ventricular arrhythmic events, and hospitalization for heart failure. Major ventricular arrhythmic events included sustained ventricular tachycardia, ventricular fibrillation, and appropriate ICD therapy. Time-to-event was defined as the interval between the index CMR examination and the first occurrence of any component of the composite endpoint. Patients without events were censored at the last available follow-up.

Exploratory analyses were also performed for arrhythmic events, and heart failure hospitalization was considered separately. The primary endpoint was defined as a composite of all-cause mortality, major ventricular arrhythmic events (sustained ventricular tachycardia or ventricular fibrillation), and hospitalization for heart failure. For statistical analyses, endpoint occurrence was coded as a binary variable (0 = no event; 1 = event).

### 2.6. Statistical Analysis

Continuous variables were expressed as mean ± standard deviation or median and interquartile range, as appropriate. Categorical variables were expressed as counts and percentages. Between-group comparisons were performed using Student’s *t*-test or the Mann–Whitney U test for continuous variables and Fisher’s exact test for categorical variables, given the limited sample size.

Kaplan–Meier survival analysis was used to estimate event-free survival, with comparisons performed using the log-rank test. Cox proportional hazards regression was used to assess associations between clinical and CMR variables and the composite endpoint. Native T1 was analyzed as a continuous variable, reported per 10 ms increment, and as a categorical variable using ROC-derived thresholds. ROC analysis was used to explore the discriminatory performance of native T1 for the composite endpoint, and the optimal cut-off was identified using the Youden index.

To explore the potential role of tissue characterization markers in risk characterization, hierarchical Cox models were constructed by sequentially adding CMR variables: Model 1 included LVEF; Model 2 included LVEF and LGE; and Model 3 included LVEF, LGE, and septal native T1. Given the limited number of events, multivariable analyses were considered exploratory and restricted to clinically relevant imaging variables. Statistical significance was defined as a two-sided *p*-value < 0.05.

Continuous variables were analyzed both as continuous measures and, when appropriate, categorized according to ROC-derived thresholds or cohort-based distributions [17,18,19]. Given the limited sample size and the relatively small number of events, Cox regression and hierarchical models were intentionally restricted to a limited number of clinically relevant imaging variables in order to reduce the risk of model overfitting. Accordingly, all regression analyses should be interpreted as exploratory and hypothesis-generating. No formal sample size calculation was performed because of the retrospective single-center design and the inclusion of consecutive eligible patients during the predefined study period. Therefore, the study was designed to identify potential associations and generate hypotheses rather than to provide definitive estimates of prognostic effect size.

## 3. Results

### 3.1. Baseline Clinical and CMR Characteristics

The final study population included 45 patients with non-ischemic DCM. The mean age was 58.1 ± 10.5 years, and 36 patients were male (80.0%). Twenty-two patients (48.9%) were referred as inpatients. NYHA class III–IV symptoms were present in 13 patients (28.9%). Hypertension was present in 23 patients (51.1%), diabetes in 13 (28.9%), dyslipidemia in 13 (28.9%), and active or previous smoking in 9 (20.0%).

Mean LVEF was 41.7 ± 13.8%, mean LVEDVi was 120.6 ± 28.9 mL/m^2^, mean LVESVi was 73.1 ± 32.8 mL/m^2^, and mean RVEF was 53.4 ± 11.5%. LGE was present in 31 patients (68.9%). Among LGE-positive patients, the most frequent pattern was mid-wall enhancement, and the most frequent location was septal involvement. Mean septal native T1 was 1038.7 ± 31.9 ms. Thirty-one patients (68.9%) exhibited septal native T1 values above the institutional upper reference limit (1017 ms; 970 ± 47 ms, mean + 1 SD). Quantitative LGE extent was not incorporated into the primary analyses because complete measurements were not available for all patients (Table 1).

### 3.2. Clinical Outcomes

During a median follow-up of 15 months, 14 patients (31.1%) reached the composite endpoint, which included all-cause mortality, major ventricular arrhythmic events, and heart failure hospitalization. Because the number of events within each endpoint component was limited, separate Cox regression analyses were not considered statistically reliable. Therefore, all survival and regression analyses were performed using the predefined composite endpoint, consistent with the exploratory nature of the study.

### 3.3. Native T1 and Clinical Outcomes

Patients who experienced the composite endpoint showed higher septal native T1 values compared with event-free patients who experienced the composite endpoint showed numerically higher septal native T1 values than event-free patients among those with available T1 measurements (1047.5 ± 25.0 ms vs. 1031.5 ± 24.3 ms), although the difference did not reach conventional statistical significance (*p* = 0.065). Accordingly, these findings should be interpreted as exploratory. ROC analysis identified a septal native T1 threshold of 1042 ms, with moderate discriminatory performance for the composite endpoint (AUC = 0.70). Given the limited sample size and number of events, this threshold should be considered hypothesis-generating and was used exclusively for descriptive graphical stratification and exploratory analyses.

### 3.4. LGE Status and Native T1

**Figure 3:**
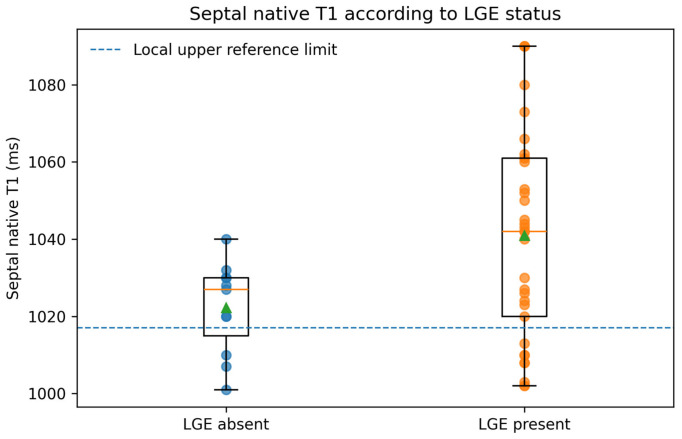
Septal native T1 values according to LGE status. Box-and-whisker plots showing the distribution of septal native T1 values in patients with and without LGE. Individual patient values are displayed as circles (blue: LGE absent; orange: LGE present). Green triangles indicate mean values for each group. The dashed horizontal line represents the institutional upper reference limit for septal native T1 (1017 ms).

**Figure 4:**
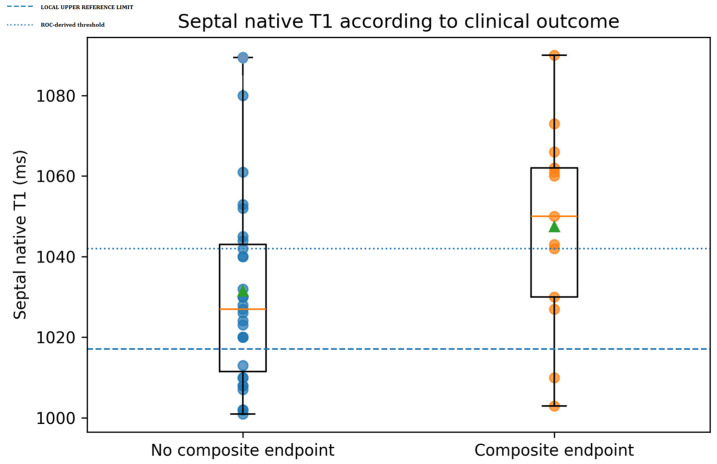
Septal native T1 values according to clinical outcome. Distribution of septal native T1 values stratified according to the occurrence of the composite endpoint during follow-up. Patients experiencing adverse clinical events tended to demonstrate higher septal native T1 values. Given the limited sample size, this analysis should be interpreted as exploratory. Blue circles represent patients without the composite endpoint, whereas orange circles represent patients who reached the composite endpoint. Green triangles indicate group mean values. The dashed horizontal line represents the institutional upper reference limit for septal native T1 (1017 ms), and the dotted horizontal line represents the exploratory ROC-derived threshold (1042 ms).

LGE was frequently observed in the study cohort and remained the principal marker of focal replacement fibrosis. In contrast, native T1 mapping was explored as a marker of diffuse myocardial abnormality; therefore, the two techniques were considered complementary measures of myocardial tissue characterization [20]. However, overlap between LGE status and septal native T1 values was observed, indicating that focal replacement fibrosis and diffuse myocardial abnormalities may provide complementary information. In selected LGE-negative patients, septal native T1 values were above the local reference range, supporting the potential role of mapping in detecting diffuse myocardial involvement not captured by conventional LGE imaging (Figure 3 and Figure 4).

### 3.5. Survival Analysis and ROC-Derived Threshold

**Figure 5:**
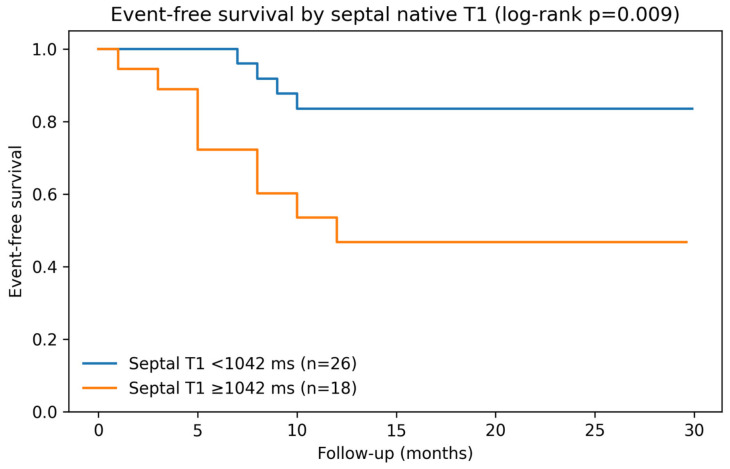
Kaplan–Meier analysis according to the septal native T1 threshold. Kaplan–Meier event-free survival according to septal native T1. Patients were stratified using the exploratory threshold of 1042 ms derived from ROC analysis. Although a numerical separation of the survival curves was observed, these findings should be considered exploratory and hypothesis-generating because of the limited sample size and number of events. Shaded areas represent 95% confidence intervals.

**Figure 6:**
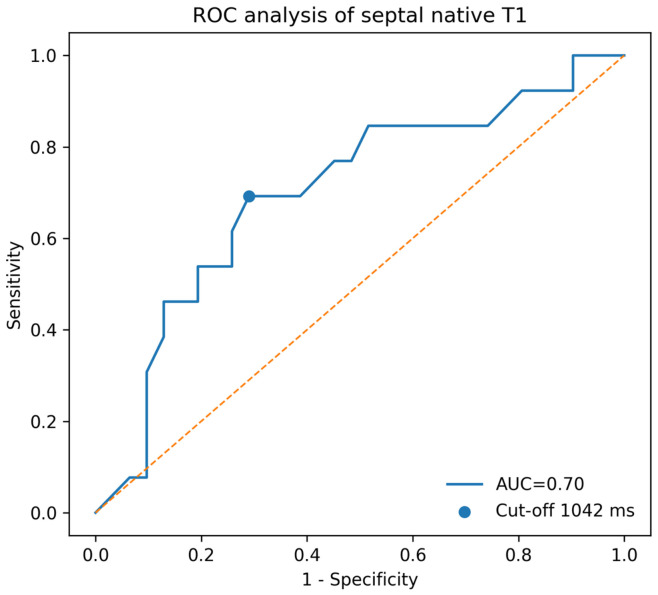
Receiver operating characteristic (ROC) curve evaluating the exploratory discriminatory performance of septal native T1 for the composite endpoint. The blue curve represents the ROC analysis, and the blue marker identifies the exploratory threshold of 1042 ms derived from the Youden index. The dashed diagonal line represents the line of no discrimination (AUC = 0.50). Given the limited sample size and number of events, ROC findings should be interpreted as hypothesis-generating.

ROC analysis identified a septal native T1 threshold of 1042 ms for prediction of the composite endpoint, with an exploratory AUC of 0.70. Kaplan–Meier analysis showed lower event-free survival in patients with septal native T1 ≥ 1042 ms compared with those below this threshold (log-rank *p* = 0.009). Given the limited sample size and number of events, these findings should be considered exploratory and hypothesis-generating rather than definitive evidence of prognostic association (Figure 5 and Figure 6).

### 3.6. Exploratory Cox Regression and Hierarchical Risk Characterization

In exploratory Cox regression analyses, functional parameters, particularly LVEF and LVEDVi, showed the strongest association with the composite endpoint. Septal native T1, analyzed both as a continuous variable and according to the ROC-derived threshold, did not demonstrate a statistically significant association with the composite endpoint and should therefore be interpreted cautiously. Hierarchical models were used exclusively for exploratory risk characterization. Model 1 included LVEF, Model 2 added LGE presence, and Model 3 added septal native T1 ≥ 1042 ms. Given the limited sample size and number of events, these models were not intended to provide evidence of incremental prognostic value, but rather to explore the potential relationship between diffuse myocardial tissue characteristics and clinical outcomes (Table 2).

In univariable analyses, LVEF and LVEDVi demonstrated the strongest associations with the composite endpoint. Septal native T1, whether analyzed as a continuous variable or according to the exploratory threshold of 1042 ms, did not demonstrate a statistically significant association with the composite endpoint. Similarly, LGE presence was not significantly associated with the composite endpoint in this cohort. Because of the limited number of events and the width of some confidence intervals, these estimates should be interpreted as exploratory and hypothesis-generating rather than definitive measures of prognostic effect. Hierarchical models were used exclusively to explore the potential relationship between diffuse myocardial tissue characteristics and clinical outcomes in conjunction with conventional functional and tissue markers. These models were not intended to establish incremental prognostic value (Table 3).

**Figure 7:**
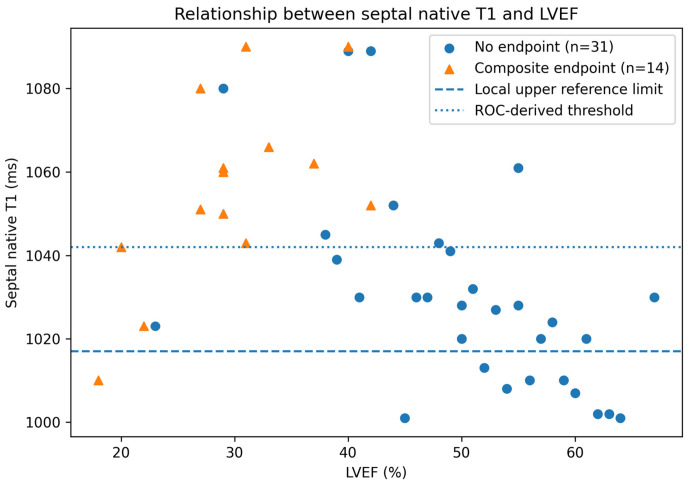
Relationship between septal native T1 and left ventricular ejection fraction (LVEF). Scatter plot illustrating the distribution of septal native T1 values according to LVEF. The horizontal dashed line indicates the institutional upper reference limit for septal native T1 (1017 ms). Patients who experienced the composite endpoint are identified separately. This figure is presented for descriptive purposes and should be interpreted in the context of the limited sample size and number of events.

**Figure 8:**
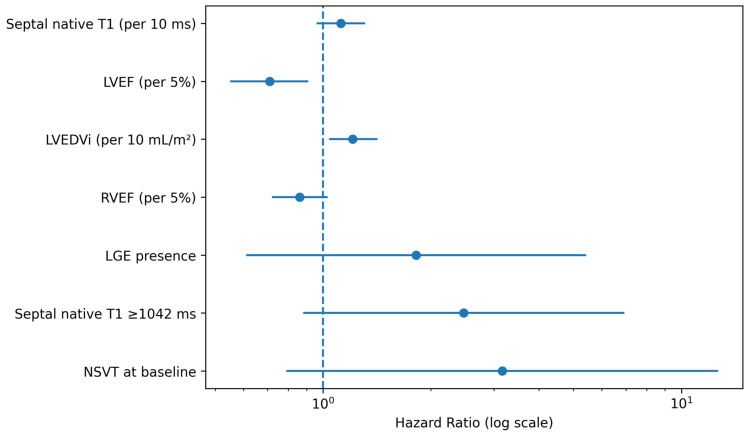
Exploratory univariable Cox regression analysis. Forest plot summarizing hazard ratios (HRs) and 95% confidence intervals for selected clinical and cardiac magnetic resonance variables associated with the composite endpoint. Functional parameters, particularly LVEF and LVEDVi, showed the strongest associations with adverse outcomes. Septal native T1 demonstrated a directionally consistent association with risk but did not reach statistical significance. The dashed vertical line indicates the null value (HR = 1.0). Given the limited number of events, all estimates should be interpreted as exploratory and hypothesis-generating.

Models were constructed hierarchically by sequentially adding tissue characterization markers to functional assessment. Given the limited number of events, these analyses should be interpreted as exploratory and hypothesis-generating (Figure 7 and Figure 8).

## 4. Discussion

In this retrospective single-center cohort of patients with non-ischemic DCM, patients who reached the composite endpoint exhibited more advanced left ventricular remodeling, lower systolic function, and numerically higher septal native T1 values. Following correction of endpoint coding and complete reanalysis of the dataset, septal native T1 did not demonstrate a statistically significant association with the composite endpoint and should therefore be interpreted cautiously. Given the limited sample size and number of events, all findings should be considered exploratory and hypothesis-generating. Nevertheless, these results support further investigation of native T1 mapping as part of a multiparametric CMR-based approach to myocardial tissue characterization and clinical risk assessment in non-ischemic DCM. This concept is highly relevant for CMR-based risk stratification, as tissue characterization may capture disease expression beyond conventional functional impairment [21,22,23,24,25]. This study suggests that native T1 mapping may reflect diffuse myocardial abnormalities associated with adverse clinical outcomes and may complement conventional CMR parameters such as LVEF and LGE. In the present cohort, septal native T1 values were numerically higher among patients experiencing adverse clinical events, and ROC analysis identified an exploratory threshold of 1042 ms. However, the discriminatory performance was moderate and should be interpreted cautiously, given the limited sample size and number of events. Accordingly, these findings should be considered exploratory and hypothesis-generating rather than evidence of a validated prognostic threshold. Further studies in larger cohorts are warranted to clarify the potential role of septal native T1 within a multiparametric CMR-based approach to myocardial tissue characterization and clinical risk assessment in non-ischemic DCM [26,27,28,29,30].

The main implication of this study is that risk in non-ischemic DCM should not be conceptualized exclusively as a function-based construct. Rather, adverse outcomes appear to reflect the interaction between ventricular dysfunction, focal replacement fibrosis, and diffuse myocardial disease, the latter being captured by native T1 mapping. This concept is aligned with the contemporary phenotype-based approach to cardiomyopathies, in which morphology, function, tissue characterization, clinical presentation, and underlying substrate are integrated to define disease expression and risk [31,32,33,34,35].

LVEF remains a key clinical parameter in DCM, but it does not directly describe myocardial tissue composition. A severely reduced LVEF may identify advanced mechanical dysfunction, but it cannot distinguish whether the underlying substrate is dominated by focal scar, diffuse fibrosis, active inflammation, edema, or potentially reversible myocardial injury. Conversely, patients with less severely reduced LVEF may still harbor a high-risk myocardial substrate. This limitation explains why LVEF alone has incomplete discriminatory power for adverse events, particularly arrhythmic outcomes.

LGE imaging has substantially improved risk stratification in non-ischemic DCM by identifying focal replacement fibrosis. Mid-wall septal LGE and complex scar distributions have been associated with increased risk of sudden cardiac death and adverse outcomes. However, LGE remains a focal scar imaging technique. It relies on relative differences in gadolinium distribution between abnormal and apparently normal myocardium and may therefore underestimate diffuse interstitial remodeling. This limitation is particularly relevant in non-ischemic DCM, where diffuse myocardial disease may be present even in the absence of a visually detectable focal scar [36,37,38,39,40].

Native T1 mapping addresses this limitation by providing a quantitative assessment of intrinsic myocardial relaxation properties before contrast administration. Elevated native T1 values may reflect diffuse interstitial fibrosis, extracellular matrix expansion, edema, and myocyte injury. Although native T1 is not histologically specific, it captures a biologically meaningful myocardial signal that appears strongly linked to clinical outcome. In the present cohort, septal native T1 values were higher in patients experiencing adverse clinical events. In the present cohort, septal native T1 values were numerically higher among patients experiencing adverse clinical events, and ROC analysis identified an exploratory threshold of 1042 ms. However, the discriminatory performance was moderate and should be interpreted cautiously, given the limited sample size and number of events. Accordingly, these findings should be considered exploratory and hypothesis-generating rather than evidence of a validated prognostic threshold. Further studies in larger cohorts are warranted to clarify the potential role of septal native T1 within a multiparametric CMR-based assessment of myocardial tissue characteristics in non-ischemic DCM.

A particularly relevant finding is the complementary relationship between LGE and native T1. LGE identifies focal replacement fibrosis, whereas native T1 captures diffuse myocardial abnormalities. These techniques should therefore not be interpreted as competing biomarkers but as different expressions of myocardial disease. In practical terms, LVEF describes global function, LGE identifies focal scar, and native T1 quantifies diffuse tissue abnormality. Their integration provides a more complete CMR phenotype of non-ischemic DCM.

The prognostic role of native T1 in LGE-negative patients is clinically important. In these patients, conventional tissue characterization may appear unremarkable despite the presence of diffuse myocardial remodeling. Elevated native T1 may therefore reveal disease that is invisible to LGE and may help identify patients who would otherwise be considered at lower risk. This observation is consistent with prior studies showing that T1 mapping and extracellular volume assessment provide prognostic information in DCM, particularly in patients without overt LGE [41,42,43,44,45].

From a methodological perspective, native T1 interpretation requires caution. T1 values are influenced by scanner platform, field strength, sequence type, acquisition parameters, and post-processing strategy. Therefore, universal cut-offs should not be applied without validation. In this study, native T1 values were interpreted in relation to the local reference range of 970 ± 47 ms. The ROC-derived threshold of 1042 ms should be considered exploratory and internally valid for this dataset rather than immediately generalizable across centers [46,47,48]. This reinforces the importance of scanner- and sequence-specific reference values in CMR mapping research [49,50].

The strengths of this study include the analysis of a consecutive clinical cohort, the use of a standardized multiparametric CMR protocol, the integration of functional and tissue characterization variables, and the evaluation of native T1 within an exploratory multiparametric risk characterization framework. The study also reflects real-world CMR practice using a Siemens 1.5-T platform and ARGUS post-processing, supporting the feasibility of native T1 mapping in routine clinical workflows.

Several limitations should be acknowledged. First, this was a retrospective single-center study with a limited sample size (*n* = 45) and a limited number of events (*n* = 14), reducing statistical power and increasing the risk of model instability and overfitting. Accordingly, Cox regression and hierarchical analyses should be considered exploratory and hypothesis-generating. Second, the median follow-up duration of 15 months may not fully capture the long-term prognostic implications of diffuse myocardial remodeling, particularly with respect to mortality. Third, detailed information regarding contemporary heart failure therapies (including ARNI, SGLT2 inhibitors, beta-blockers, and mineralocorticoid receptor antagonists) was not consistently available and therefore could not be incorporated into the analyses, leaving the possibility of residual confounding. Fourth, the underlying etiology of DCM was not systematically available in all patients; genetic, inflammatory, idiopathic, and tachycardia-induced forms may exhibit distinct myocardial tissue characteristics and clinical trajectories. Finally, native T1 values are scanner-, sequence-, and post-processing-dependent; therefore, the proposed 1042 ms threshold should be regarded as internally derived and exploratory and requires external validation before broader application [51,52,53].

Despite these limitations, the present findings support the integration of native T1 mapping into multiparametric CMR assessment of non-ischemic DCM. Native T1 may help shift risk stratification from a purely function-based model toward a tissue-based approach, in which diffuse myocardial disease is recognized as a relevant determinant of prognosis.

## 5. Conclusions

Septal native T1 values were numerically higher in patients experiencing adverse clinical outcomes and may reflect diffuse myocardial remodeling in non-ischemic dilated cardiomyopathy. However, septal native T1 was not independently associated with the composite endpoint in exploratory Cox analyses, and the ROC-derived threshold demonstrated only moderate discriminatory performance. Therefore, the present findings should be interpreted as exploratory and hypothesis-generating.

These results support further investigation of native T1 mapping within a broader multiparametric CMR-based assessment of non-ischemic DCM, integrating ventricular function, focal fibrosis, and quantitative tissue characterization. Risk assessment in non-ischemic DCM should not rely exclusively on systolic function, but may benefit from a comprehensive tissue-based imaging approach.

Given the retrospective single-center design, limited sample size, limited number of events, and incomplete availability of etiological and treatment-related data, no definitive conclusions regarding the independent prognostic significance of native T1 can be drawn. Larger prospective multicenter studies with longer follow-up, standardized mapping protocols, systematic etiological characterization, and detailed information on contemporary heart failure therapy are required to clarify the relationship between native T1 mapping and clinical outcomes and to better define its potential role in clinical risk assessment.

## Figures and Tables

**Table 1:** Baseline clinical and CMR characteristics of the study population (*n* = 45). Values are presented as mean ± standard deviation, median (interquartile range), or counts and percentages, as appropriate. CMR = cardiac magnetic resonance; LVEF = left ventricular ejection fraction; LVEDVi = left ventricular end-diastolic volume index; LVESVi = left ventricular end-systolic volume index; RVEF = right ventricular ejection fraction; LGE = late gadolinium enhancement; NSVT = non-sustained ventricular tachycardia; ICD = implantable cardioverter-defibrillator. Percentages are based on the overall study cohort unless otherwise specified. Missing values were not imputed.

Variable	Overall Cohort (*n* = 45)
Age, years	58.1 ± 10.5
Male sex, *n* (%)	36 (80.0)
Inpatient at CMR, *n* (%)	22 (48.9)
NYHA class III–IV, *n* (%)	13 (28.9)
Hypertension, *n* (%)	23 (51.1)
Diabetes mellitus, *n* (%)	13 (28.9)
Dyslipidemia, *n* (%)	13 (28.9)
Current/former smoking, *n* (%)	9 (20.0)
Syncope, *n* (%)	3 (6.7)
NSVT at baseline, *n* (%)	4 (8.9)
LVEF, %	41.7 ± 13.8
LVEDVi, mL/m^2^	120.6 ± 28.9
LVESVi, mL/m^2^	73.1 ± 32.8
LV mass index, g/m^2^	90.0 ± 28.0
RVEF, %	53.4 ± 11.5
Septal native T1, ms	1038.7 ± 31.9
Septal native T1 >1017 ms, *n* (%)	31 (68.9)
LGE present, *n* (%)	31 (68.9)
Mid-wall LGE, *n* (%)	14 (31.1)
Subepicardial LGE, *n* (%)	6 (13.3)
Insertion-point LGE, *n* (%)	3 (6.7)
Mixed/non-ischemic LGE, *n* (%)	8 (17.8)
Septal LGE, *n* (%)	13 (28.9)
Lateral LGE, *n* (%)	7 (15.6)
Inferior LGE, *n* (%)	4 (8.9)
Anterior LGE, *n* (%)	2 (4.4)
Multiple LGE locations, *n* (%)	5 (11.1)
Follow-up duration, months	15 (IQR 10–18)
Composite endpoint, *n* (%)	14 (31.1)
ICD implantation, *n* (%)	15 (33.3)
Appropriate ICD therapy, *n* (%)	9 (20.0)

**Table 2:** Exploratory univariable associations with the composite endpoint. Hazard ratios (HRs) are reported with 95% confidence intervals. Functional parameters, particularly LVEF and LVEDVi, showed the strongest associations with the composite endpoint. Septal native T1 and LGE presence did not demonstrate statistically significant associations in this exploratory cohort. Given the limited sample size and number of events, these findings should be interpreted as hypothesis-generating rather than definitive estimates of prognostic effect size.

Predictor	HR	95% CI	*p*-Value
Septal native T1 (per 10 ms increase)	1.12	0.96–1.31	0.15
LVEF (per 5% increase)	0.71	0.55–0.91	0.008
LVEDVi (per 10 mL/m^2^ increase)	1.21	1.04–1.42	0.015
RVEF (per 5% increase)	0.86	0.72–1.03	0.10
LGE presence	1.82	0.61–5.42	0.28
Septal native T1 ≥ 1042 ms	2.47	0.88–6.94	0.087
NSVT at baseline	3.16	0.79–12.67	0.10

**Table 3:** Exploratory hierarchical models. Models were constructed sequentially using LVEF, LGE presence, and septal native T1 to characterize the distribution of functional and tissue-related variables within the study cohort. Because of the limited sample size and number of events, these analyses were performed for descriptive and exploratory purposes only and should not be interpreted as evidence of independent or incremental prognostic value.

Model	Variables Included	Model χ^2^	Interpretation
Model 1	LVEF	2.1	Functional impairment was associated with adverse clinical outcomes.
Model 2	LVEF + LGE	3.3	Addition of focal fibrosis improved characterization of myocardial substrate.
Model 3	LVEF + LGE + Septal native T1 ≥ 1042 ms	4.5	Addition of native T1 suggested potential complementary information regarding diffuse myocardial abnormalities.

## Data Availability

The data that support the findings of this study are available from the corresponding author upon reasonable request, in accordance with institutional policies and applicable regulations.
